# Mr. Harold, on Cold Water in Burns and Scalds

**Published:** 1806-05

**Authors:** E. Harrold

**Affiliations:** Cheshunt, Herts


### *To the Editors of the Medical and Physical Journal.*

GENTLEMEN,

I Am very glad to see in your last number that Dr. Kinglake has taken up the subject of burns; it certainly is a subject of great importance, and demands very serious attention. I wish to preface my reply to Dr. Kinglake by stating, that I have not the slightest knowledge of Doctor Kentish, except from his writings; that before their publication I had too frequently seen the ill effect of cold applications, even that of *cold water,* in burns and scalds, not to wish for a more efficacious mode of treatment; that I do not suppose the *facts* advanced by Dr. Kentish, who appears to have had ample experience in those cases, can be fairly questioned by any one who has not repeated his experiments; and that having seen the happy effect of the treatment recommended by him in the case which I have recorded in your Journal, and which I think was a very serious case, I felt myself justified in giving it to the world.

I am aware that medical facts are by no means easily appreciated, and that therefore it is highly praiseworthy to advance them with modesty, and to reason upon them with care. The laws of excitability are highly curious and interesting, and the Brunonian theory seems to be the first step securely taken towards their developement; but the peculiar sensibilities of particular parts of the body, their relative importance, and their sympathetic influences, demand long and patient investigation. If any man will be hardy enough to reason against facts, I do not see upon what ground he can be met. I *must* suppose that the principal object of a liberal writer is an inquiry after truth. Dr. Kinglake has I think been attacked unfairly; but why should he employ the same unfair weapon? The evidence of a respectable man ought not to be questioned until the experiment has failed in the hands of another; for, after all, experience must decide, and every mode of reasoning must prove futile without its support. I cannot compliment Dr. Kinglake on his analogy of a *runaway horse,* it appears to me to be infinitely more fanciful than just, and to have no other relation to the subject in question than matter has to mind. Dr. Kinglake goes on to remark, that "the theory which prescribes the reconducting of inflammatory disease to the standard of health, health, by only gradually diminishing the force of the violence which occasioned it, is, in my apprehension, fraught with gangrene and death." But experience has proved that this apprehension, with respect to burns, is groundless; and the Doctor has here evidently confounded the violence of the exciting cause with the violence of the consequent action. As experience has proved the efficacy of the *gradual restoration* to the healthy degree of excitement, from one extremity of the scale of excitability, so has it also from the other. I do not think Dr. Kinglake would be hardy enough to plunge a frozen limb into *hot* or even *warm* water, for experience has most decidedly proved that such a measure would be productive of the "gangrene and death" of the part. And if the Doctor should ride his fiery steed so temperately in a very cold day, as to find when he enters a warm room, his fingers extremely painful; the instantaneous relief afforded by rubbing them with a little snow might serve to convince him, that a very gradual restoration of heat is not only the most agreeable but the best practice; for the too sudden application of heat does not merely occasion great pain, but that pain is followed by a considerable degree of numbness.

Dr. Kinglake then observes, "If, pursuant to the Brunonian doctrine of reducing excitement only by a slow series of diminution, be the correct method of treating diseases of indirect debility induced by excessive stimulation, when can a warranty arise for copious bleeding," &c. &c. and answers the question thus: "under no circumstances whatever." Here the question is again involved in a difficulty, which I confess surprises me. Surely, there is a wide difference between the *transient* application of a powerful exciting cause and one that *continues* to operate with undiminished force. I am disposed to think, that if we had such morbid causes as compleatly within our power as we have that of the external application of heat; by gradually withdrawing them we should cure our patients more speedily, and with less ultimate debility, than by the indirect mode of evacuations. He then says, "the diseases of *direct* debility will imperiously demand liberal excitement." Now this doctrine is any thing rather than Brunonian, for, according to that system, diseases of direct debility are to be cured by the most *gradual* application of stimulants, or, as the author expresses himself, by "gradually wearing down the accumulated excitability." I have said thus much, gentlemen, in support of a theory which is not my own, but which I think fairly bears me out, and explains the facts I believe better than any other; but as my anxiety is much more to discover and establish truth than to theorize; and as I think that a calm investigation is infinitely better calculated to lead to that discovery, than an intemperate inquiry; if you should perceive any undue asperity in this letter, I beg that you will correct it. I also request Dr. Kinglake to accept my thanks for entering upon the subject.

I am, &c.

*Cheshunt, Herts, April* 2, 1806. E. HARROLD.

